# Pre-Clinical Evaluation of rHDL Encapsulated Retinoids for the Treatment of Neuroblastoma

**DOI:** 10.3389/fped.2013.00006

**Published:** 2013-03-21

**Authors:** Nirupama Sabnis, Suraj Pratap, Irina Akopova, Paul W. Bowman, Andras G. Lacko

**Affiliations:** ^1^Molecular Biology/Immunology, University of North Texas Health Science CenterFort Worth, TX, USA; ^2^Pediatrics, SUNY Downstate Medical CenterBrooklyn, NY, USA; ^3^Pediatrics, University of North Texas Health Science CenterFort Worth, TX, USA

**Keywords:** drug delivery, nanoparticles, rHDL, neuroblastoma, fenretinide, all-trans-retinoic acid

## Abstract

Despite major advances in pediatric cancer research, there has been only modest progress in the survival of children with high risk neuroblastoma (NB) (HRNB). The long term survival rates of HRNB in the United States are still only 30–50%. Due to resistance that often develops during therapy, development of new effective strategies is essential to improve the survival and overcome the tendency of HRNB patients to relapse subsequent to initial treatment. Current chemotherapy regimens also have a serious limitation due to off target toxicity. In the present work, we evaluated the potential application of reconstituted high density lipoprotein (rHDL) containing fenretinide (FR) nanoparticles as a novel approach to current NB therapeutics. The characterization and stability studies of rHDL-FR nanoparticles showed small size (<40 nm) and high encapsulation efficiency. The cytotoxicity studies of free FR vs. rHDL/FR toward the NB cell lines SK-N-SH and SMS-KCNR showed 2.8- and 2-fold lower IC_50_ values for the rHDL encapsulated FR vs. free FR. More importantly, the IC_50_ value for retinal pigment epithelial cells (ARPE-19), a recipient of off target toxicity during FR therapy, was over 40 times higher for the rHDL/FR as compared to that of free FR. The overall improvement in *in vitro selective* therapeutic efficiency was thus about 100-fold upon encapsulation of the drug into the rHDL nanoparticles. These studies support the potential value of this novel drug delivery platform for treating pediatric cancers in general, and NB in particular.

## Introduction

Neuroblastoma (NB) is one of the most frequently diagnosed tumors in pediatric patients. The wide spectrum of its clinical presentation presents a major therapeutic challenge (1). Recently, a stratification strategy for NB cases has emerged based on established clinical and biological criteria as a basis for designing effective therapeutic regimens for the specific forms of the disease (2). According to this strategy, approximately 40% of NB patients are classified as having the high risk form of the disease (HRNB) (2, 3).

Current treatment of NB patients is largely dependent on the location of the tumor, the stage of the disease, and the age of the patient (4, 5). In certain localized cases, surgery alone is sufficient; however, most often additional therapeutic interventions, including chemotherapy, radiation, and autologous stem cell therapy are also employed, especially if metastatic lesions are present (2, 3). The HRNB variant is extremely resistant to the currently available treatment regimens and often results in relapses subsequent to the initial phases of the therapy (6, 7). Matthay et al. have reported that the best outcome for HRNB was achieved with intensive combination of chemotherapy and surgery, followed by myeloablative therapy with hematopoietic stem cell rescue, and then differentiation therapy with isotretinoin (8). Extensive pre-clinical and early clinical trials for treatment of NB have focused on small-molecule inhibitors targeting specific genetic pathways, implicated in the proliferation of NB cells. These include the insulin-like growth factor I receptor (IGF-IR), phosphoinositide 3-kinase (PI3K), mammalian target of rapamycin (mTOR), and Akt (9–13). An alternative approach involves immune therapy utilizing cytokine, vaccine, antibody, and cellular therapy (14–16). Despite recent advances in the development of anti-cancer agents and the use of multi-modal therapeutics for the treatment of HRNB the morbidity and mortality in this group of patients remains high (3–5, 17).

Retinoids are analogs of vitamin A, involved in the control of cell differentiation and proliferation (18). All-trans-retinoic acid (ATRA), and Fenretinide (FR) (*N*-(4-hydroxyphenyl) retinamide), a synthetic analog of ATRA, have emerged as promising tumor preventive and therapeutic agents against preneoplastic and neoplastic lesions (19–21). Both FR and ATRA are antiproliferative as well as apoptotic in *in vivo* and *in vitro* models (22, 23), including breast cancer cells (24), ovarian carcinoma cells (25), acute myeloid leukemia cells (26), and NB cells (27). However, phase I and II clinical trials have revealed some side effects, including nyctalopia (night blindness), and dermatological sensitivity (21). ATRA has been shown to promote the cellular differentiation of malignant cells and ATRA-induced remission was demonstrated in preventing relapse and maintaining remission in NB patients subsequent to intensive initial therapy (28, 29).

Overall, the current state of the art of NB therapeutics is complex and not yet fully effective, especially in case of high risk and refractory NB. Consequently, development of advanced treatment modalities is needed, especially to avoid the morbidity of toxic side effects during chemotherapy. Targeted drug delivery has been an important focus of recent research, especially for cancer chemotherapy (30–33). Numerous projects have focused on selective tumor delivery of highly toxic drugs that can be transported to cancer cells and tumors without damage to normal tissues (34–36). It has been suggested that lipoproteins have structural properties that enable them to serve as drug delivery vehicles because of their ability to incorporate hydrophobic drugs into their micellar core and subsequently facilitate the cellular uptake of drugs via a tumor selective receptor mediated mechanism (36–41). Potential of lipoproteins as the “magic bullet” for delivery of targeted chemotherapy was postulated by Counsell and Pohland over 30 years ago (42). Nevertheless, until recently lipoproteins have been largely overlooked as drug delivery agents despite their potential for receptor mediated tumor specific targeting (36, 37).

The purpose of these studies was to provide proof of concept for a novel therapeutic approach by encapsulating known anti-NB agents in a lipoprotein based formulation to achieve selectively targeted delivery of anti-cancer drugs to NB tumors. Initially, FR and ATRA were selected as representative drugs based on the physical/chemical properties, the therapeutic efficacy of these agents during pre-clinical (43, 44) and clinical studies (45, 46). FR and ATRA also exhibit an adverse pharmacokinetic profile, due to their extensive lipophilicity, thus limiting their systemic application (47). To increase their bioavailability, we incorporated ATRA and FR into reconstituted high density lipoprotein (rHDL) nanoparticles and subsequently evaluated some physical and chemical properties and the anti-NB potential of the resultant drug formulation.

## Materials

Sodium cholate, egg yolk phosphatidylcholine (PC), free cholesterol (FC), cholesteryl oleate, potassium bromide (KBr), isopropylthiogalactoside (IPTG), dimethyl sulfoxide (DMSO), phenylmethylsulfonyl fluoride (PMSF), Triton X-100, thrombin cleavage kit, FR, and ATRA were purchased from Sigma-Aldrich Corporation, St Louis, MO, USA. NZYCM was obtained from Teknova, Hollister, CA, USA. Bacterial protein extraction reagent and bicinchoninic acid (BCA) protein assay kits were purchased from Thermo Scientific, Rockford, IL, USA. A histidine-trap (His-Trap) affinity column was obtained from QIAGEN, Valencia, CA, USA. Cholesterol and phospholipid estimation kits were obtained from Wako Pure Chemical Industries Ltd., Richmond, VA, USA. Block lipid transport-1 (BLT-1) was obtained from Cambridge Corporation (San Diego, CA, USA) and prepared as 5 mg/ml stock solution in 100% DMSO. Roswell Park Memorial Institute (RPMI) 1640 media, Iscove’s Modified Dulbecco’s media (IMDM), and fetal bovine serum (FBS) were obtained from Invitrogen, Carlsbad, CA USA.

The NB cell lines SK-N-SH and SMS-KCNR were obtained from Children’s Oncology Group (COG) Cell Culture/Xenograft Repository, Texas Tech., Lubbock, TX, USA. A retinal pigment epithelial cell line ARPE-19 was obtained from American Type Culture Collection (ATCC).

## Methods

### Preparation of the ATRA and FR containing nanoparticles

#### Isolation and purification of recombinant apoA-I

These were performed by the procedure already established (48). Briefly, BL21(DE3)pLysS cells bearing the pNFXex plasmid were cultured in 500 ml NZYCM media containing 50 μg/ml ampicillin at 37°C. Upon reaching the culture optical density to 0.6 at 600 nm, ApoA-I synthesis was induced by the addition of IPTG to a final concentration of 0.5 mM. After 3 h, the bacteria were pelleted by centrifugation and disrupted by lysis buffer (bacterial protein extraction reagent). The cell lysate was centrifuged at 20,000 *g* for 30 min at 4°C. The supernatant fraction was mixed with an equal volume of phosphate-buffered saline (PBS) containing 6 M guanidine hydrochloric acid (Gn-HCl), applied to a 5 ml bed volume His-Trap affinity column, and purified as per manufacturer’s instructions. The N-terminal His-Tag extension was removed using a thrombin CleanCleave kit (Sigma-Aldrich). The isolated apo A-I was then dialyzed against tris(hydroxymethyl)aminomethane (Tris)-buffered saline containing 1 mM benzamidine for 16 h at 4°C. The dialyzed sample was filter-sterilized (0.2 μm) and stored at 4°C until use.

#### Preparation of rHDL/ATRA and rHDL/FR complexes

This was accomplished by a procedure developed earlier in our laboratory (36–38, 40, 41). Briefly, a mixture of egg yolk PC in CHCl_3_ with FC, and cholesteryl oleate (CE), was prepared with a molar ratio of ApoA1:FC:CE:PC = 1:5:1.3:1.15 M. The lipid mixture (PC, FC, and CE) and the drugs (ATRA or FR) and the tracer [H]^3^ATRA were dried under nitrogen to a thin film and dispersed in 60 μl DMSO. To this mixture, apo A-I (5 mg) and 140 μl sodium cholate (from a stock of 100 mM) were added and the volume was made up to 2 ml with Tris-ethylenediaminetetraacetic acid (EDTA) buffer (10 mM Tris, 0.1 M KCl, 1 mM EDTA pH 8.0). The final PC to cholate molar ratio was maintained at (1:1.6). The lipid/protein/cholate mixture was then incubated for 12 h at 4°C, followed by dialysis against 2 l of PBS, for 48 h, with three buffer changes in the first 12 h. The preparations were then centrifuged at 1000 rpm for 2 min and sterilized using a 0.2 μm syringe filter. The preparations were kept in the dark at 4°C until used.

### Optimization of drug loading studies with rHDL/ATRA nanoparticles

Reconstituted high density lipoprotein/all-trans-retinoic acid particles were prepared using increasing initial concentrations of ATRA from 0.25 to 3 mg/ml while keeping all other ingredients constant. The percentage ATRA incorporation was determined by measuring the initial and the final radioactive counts incorporated in nanoparticles with the Tri-Carb 2100 TR Liquid Scintillation Analyzer, East Setauket, NY, USA.

The percentage drug incorporated or drug entrapment efficiency was determined using the Eq. 1:

(1) $$\begin{array}{llll} \text{DEE} & =\left\{\text{Drug}\;\text{concentration}\;\text{after}\;\text{dialysis}∕\right. & & \\ & \left.\;\text{Drug}\;\text{concentration}\;\text{before}\;\text{dialysis}\right\}\times 100 \end{array}$$

At each of the drug concentrations, the polydispersity index (PDI) and diameter of the nanoparticles was established from using data from dynamic light scattering (DLS) studies as indicated below. PDI was calculated as described earlier (49) using a span index, which was calculated as per Eq. 2.

(2) Span=(D0.9−D0.1)/D0.5

where *D*0.9, *D*0.5, and *D*0.1 are the particle diameters determined, respectively, at the 90th, 50th, and 10th percentile of the undersized particle distribution curve.

### Characterization of rHDL/ATRA and rHDL/FR nanoparticles

#### Determination of Chemical composition of the rHDL nanoparticles

Cholesterol and phospholipid contents were determined by respective enzymatic reagent kits (cholesterol E and phospholipid C), using microtiter plate assays as per manufacturer’s suggestions. Protein determinations were carried out using a BCA protein assay kit. The percentage ATRA incorporation was determined as described above. The FR incorporation efficiency was determined using the initial and final concentration of rHDL/FR formulation using absorption maxima at 340 nm in a spectrophotometer (Varian Inc., Mulgrave, Australia).

### Estimation of the size and morphology of rHDL/ATRA and rHDL/FR nanoparticles

#### Dynamic light scattering

Particle size analysis of the ATRA and FR loaded rHDL nanoparticles was carried out using a Nanotrac system (Microtrac Inc., Montgomeryville, PA, USA) as per manufacturer’s instructions. The nanoparticles were dispersed in aqueous buffer using an ultrasonic water bath (Fisher Scientific, Pittsburgh, PA, USA) for 2 min and then measured for particle size. The results were reported as the average of three independent runs with duplicate observations in each run. The PDI was calculated using Eq. 2 as described earlier.

#### Atomic force microscopy

The rHDL-ATRA and rHDL-FR formulation was dialyzed at 4°C for 12–18 h against sterile distilled and deionized water to remove the salts from the solution. It was further diluted 1:5 with sterile distilled water. Ten microliters of the sample was then placed on the microscope slide and was allowed to air dry. Sample was then processed with atomic force microscopy (AFM) on an NTEGRA Prima scanning probe microscope (NT-MDT, Santa Clara, CA, USA). Closed-loop feedback semicontact mode was used at a rate of 0.6 Hz. Scanning started from the 50 μm area, going down to 5 μm. The images obtained were analyzed with NT-MDT image analysis software (v 2.2). At least 25 individual particles were measured from three different positions and the average diameter was reported.

### Effect of free and rHDL encapsulated FR on cell viability

Culturing of the NB cell line (SK-N-SH and SMS-KCNR), and retinal pigment cell line (ARPE-19) were carried out according to procedures and culturing conditions provided by the COG and ATCC (50, 51). Briefly, the SK-N-SH cells were cultured in RPMI 1640 containing 2 mM l-Glutamine, 4500 mg/l glucose, 10 mM HEPES, 1500 mg/l Sodium bicarbonate, and 10% FBS. SMS-KCNR was grown in IMDM with 2 mM l-Glutamine, Insulin Transferrin Selenium (ITS), and 10% FBS. ARPE-19 cells were grown in RPMI 1640 containing 2 mM l-Glutamine and 10% FBS. All the cells were grown in 75 cm^2^ flasks and incubated at 37°C and 5% CO_2_. Cells were passaged using 0.25% trypsin to release the cells from the flasks, once 80–90% confluency was reached.

### Preparation of drug formulations

Both FR and ATRA stock solutions were prepared at 50 mg/ml concentration in DMSO. For cytotoxicity studies, the drugs were prepared by emulsifying in 50% sterile bovine serum albumin (BSA)/PBS by stirring. Samples for the characterization studies were prepared by diluting the stock with PBS to achieve equivalent FR molar concentration to that contained by the respective rHDL/FR nanoparticle samples. (The unencapsulated FR suspension in BSA is referred to as “free drug.”)

### Determination of IC_50_ doses

The effect of FR in free drug and rHDL encapsulated particles was studied using CCK-8 kit (Dojindo Molecular Technologies, Tabaru, Japan). Briefly, the NB cell lines SMS-KCNR and SK-N-SH were grown according to procedures and culturing conditions provided by the ATCC in the irrespective media as stated earlier in 75 cm^2^ flasks and incubated at 37°C and 5% CO_2_ (50, 51). Cells were passaged using 0.25% trypsin to release the cells from the flasks, once 80–90% confluency was reached. Cells were counted using hemocytometer and 5000 cells were seeded per well into 96-well microtiter plates and incubated at 37°C in 5% CO_2_ for 24 h to allow the cells to attach to the plates. The free drug and the rHDL/FR nanoparticles were diluted in serum-free medium to yield stock solutions of equivalent molar concentrations. Subsequently, aliquots of the stock solutions were added to microtiter plate wells to achieve the selected concentration range for the cell viability tests. Controls included cells with media (without rHDL/drug), cells with empty rHDL particles, and media without cells with same rHDL/drug and free drug of each concentration used. Cells were incubated at 37°C in 5% CO_2_ for 24 h. After incubation, 10 μL of highly water-soluble tetrazolium salt, WST-8 [2-(2-methoxy-4-nitrophenyl)-3-(4-nitrophenyl)-5-(2,4-disulfophenyl)-2H-tetrazolium, monosodium salt] stock solution (Dojindo) was added to each well. After 3 h of incubation at 37°C, the absorbance at 450 nm was measured using a Bio-Rad 3550 microplate reader (Bio-Rad Laboratories, Hercules, CA, USA). Each concentration was studied with six replicates.

### Inhibition of drug uptake from the rHDL/FR and rHDL/ATRA complexes

#### Using human HDL

Neuroblastoma cells SMS-KCNR were plated in 24-well plates (100,000 cells/well) in their respective media. On the following day, the monolayers were washed with PBS, pH 7.4, and then incubated at 37°C with serum-free medium for 90 min. Cells were washed with PBS and incubated with a single concentration of the rHDL/FR complex plus increasing amounts of HDL* (0–120 μg) in serum-free medium for 90 min. The preparation was washed once with 1 × PBS, pH 3.0 and subsequently with 1 × PBS, pH 7.4 respectively. The cells were then lysed with lysis buffer (50 mm Tris-HCl [pH 8.0], 150 mM NaCl, 0.02% sodium azide, 100 μg/ml PMSF, 1 μg/ml aprotinin, and 1% Triton X-100). The lysate was centrifuged at 10,000 rpm for 5 min. The protein content of the lysate was determined by BCA assays. The FR content and ATRA content was followed by spectrophotometric and radioactivity measurements respectively as described above. HDL* was prepared using ultracentrifugation of fresh serum in potassium bromide density gradient as reported in the literature (52).

#### Using block lipid transport-1

The inhibition of uptake of ATRA was also studied in the presence of BLT (2-Hexyl-1-cyclopentanone thiosemicarbazone) a known inhibitor of SR-B1 receptors (53). At 0.4 and 2 μM concentration of BLT in SMS-KCNR by the same procedure as described above.

### Stability studies

#### Stability of the nanoparticles at low temperature

The nanoparticles were stored at 4°C and −20°C. After a month, 100 μl aliquots were removed. The samples were dialyzed, using dialysis tubing with an 8000 MW cut off, at 4°C for 18 h. The FR content and particle size before and after dialysis were determined spectrophotometrically and by DLS methods respectively as described above.

#### Stability to lyophilization

Reconstituted high density lipoprotein/fenretinide particles were dialyzed against 0.1 M PBS. The nanoparticles were freeze-dried at −56°C/0.5 Mbar in a Labconco freeze drier (Labconco Corp., Kansas City, MO, USA) until they were completely dry, producing a powder. The freeze-dried preparations were stored at −20°C for 24 h and then reconstituted in 1 × PBS. The FR content and particle size before and after dialysis were determined spectrophotometrically and by DLS methods respectively as described above.

## Results

The aim of this project was to characterize the ATRA and FR containing rHDL nanoparticles and to evaluate the feasibility for enhancing the cytotoxic efficacy of FR against NB cells. Because of its high octanol-water coefficient (XlogP), ATRA was considered to be an appropriate candidate to be transported by the rHDL drug delivery system and a model for the incorporation of FR. The rHDL particles with ATRA were prepared as described earlier (36–38, 40, 41). With increasing the initial concentration of ATRA from 0.25 to 3 mg/ml, a steady decrease in the percentage incorporation was observed.

**Figure 1:**
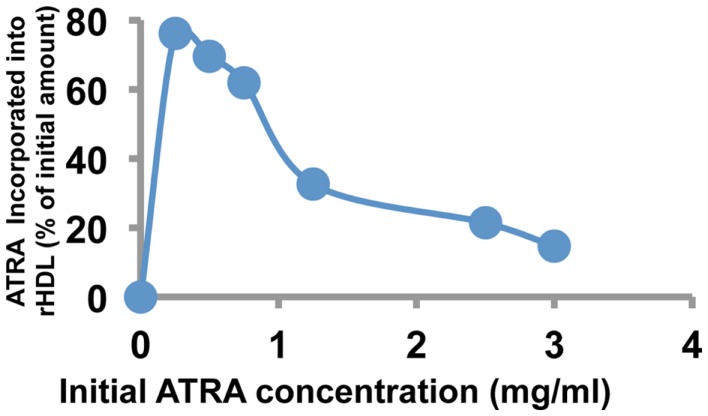
**Incorporation efficiency of ATRA into rHDL nanoparticles as a function of initial ATRA concentration**.

As shown in Figure 1. The highest ATRA incorporation efficiency of 76.12% was observed at 0.25 mg/ml where as it progressively reduced to 14.6% at the initial ATRA concentration of 3 mg/ml. The corresponding particle size at each of the respective initial drug concentrations showed that at 0.75 mg/ml the mean particle diameter was smallest (86 nm) as compared to both extreme initial concentrations of 0.25 mg/ml (102.2 nm) and 3 mg/ml (167 nm); as estimated by DLS. A similar pattern was observed with the PDI upon increase in the initial ATRA concentration where there was an initial decrease in the PDI with increase in the initial concentration of ATRA from 0.25 to 0.75 mg/ml after which the PDI increased from two to five times with further increase in the initial concentration of ATRA from 1.25 to 3 mg/ml respectively. Overall the PDI was acceptable at all particle size at ∼0.1 (considerably below the suggested desirable range: <0.2).

From these initial studies, 0.75 mg/ml initial concentration of ATRA in the rHDL nanoparticles was shown to be optimal in terms of incorporation efficiency and polydispersity. Due to the structural similarities between ATRA and FR, the same loading conditions were employed for the formulation of rHDL-FR nanoparticles in subsequent studies.

**Figure 2:**
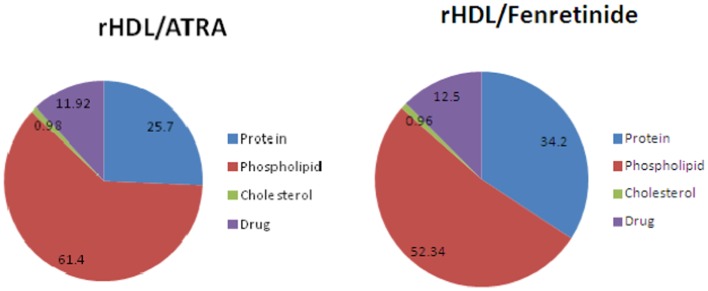
**Chemical composition of rHDL/ATRA and rHDL/FR particles**.

The chemical composition of the ATRA and FR containing rHDL nanoparticles is shown in Figure 2., The largest component of both rHDL/ATRA and rHDL/FR nanoparticles was phospholipid (61.4 and 52.34% respectively) followed by protein/apo A-I (25.7 vs. 34.2%), the respective drugs and cholesterol. The rHDL/ATRA particles had an ∼12% ATRA content and the rHDL/FR nanoparticles a 12.5% FR content, similar to earlier findings with paclitaxel containing rHDL nanoparticles (38, 40, 41).

**Figure 3:**
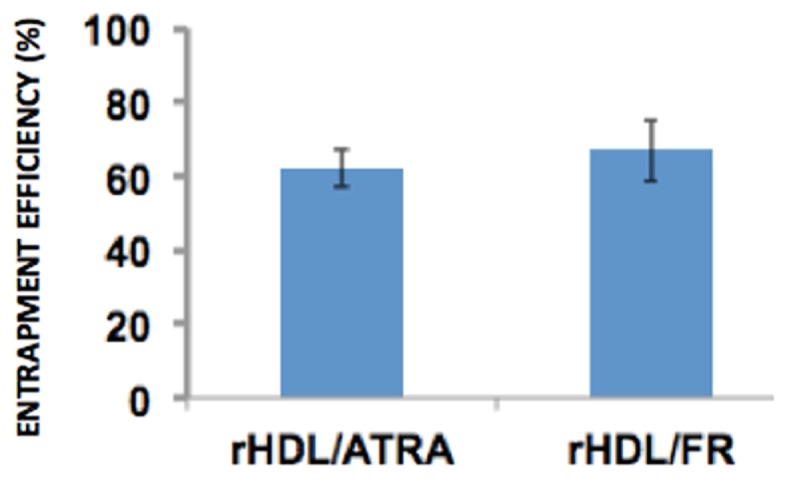
**Drug entrapment efficiency of rHDL/ATRA and rHDL/FR nanoparticles**.

The entrapment of efficiency of both rHDL/ATRA and rHDL/FR nanoparticles were 61.95 and 67.07% respectively (Figure 3) suggesting that ATRA and FR could both be efficiently incorporated into rHDL nanoparticles.

**Figure 4:**
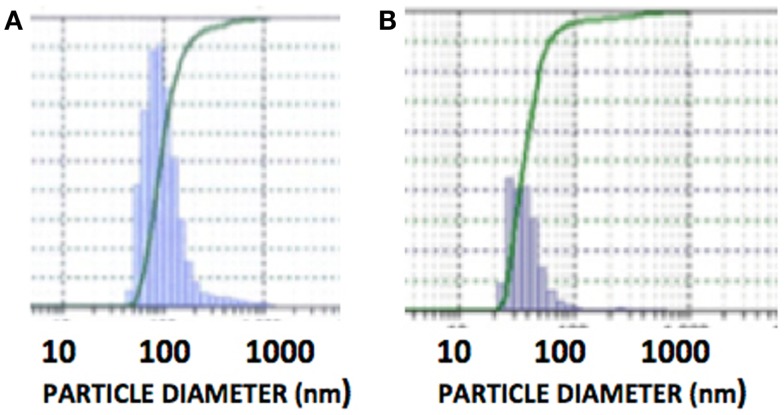
**Size distribution of rHDL/ATRA (A) and of rHDL/FR (B) nanoparticles with DLS**.

**Figure 5:**
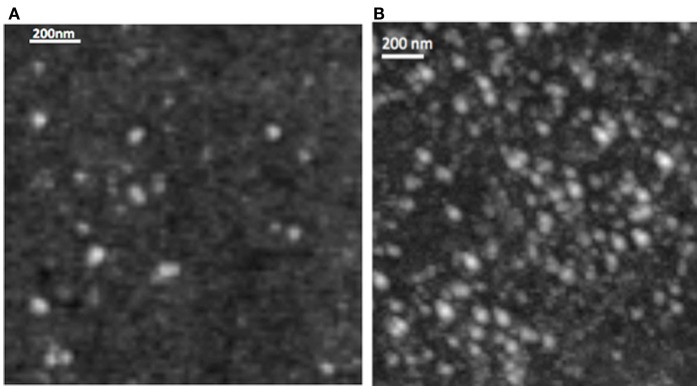
**Morphology of rHDL/ATRA (A) and of rHDL/FR (B) nanoparticles with Atomic Force Microscopy**.

The DLS measurement of the size distribution of retinoid nanoparticles are presented in Figures 4A,B. The size of rHDL/ATRA ranged from 48 to 91 nm with average of 86 nm. The rHDL/FR particles were found to be distributed in a narrower range of 22–101.2 nm with average diameter of 38.3 nm. AFM analysis of the rHDL-ATRA (A) and rHDL-FR (B) nanoparticles (Figure 5) was consistent with the shape and size distribution of the particles. The particles were found to be spherical in shape with the respective mean diameters of 8 and 15 nm for rHDL-ATRA and rHDL-FR particles, close to values reported for native HDL (54). The discrepancy between the observed diameters (22 and 86 nm) by DLS vs. 8 and 15 by AFM may be attributed to the tendency to select zones of clear, uniformly distributed particles while the larger aggregated particles may be missed with AFM, while, DLS tends to yield larger particle diameter figures, due to well known bias for the larger diameter particles (Figures 4A,B). In addition, the discrepancy between the size estimates by DLS vs. AFM is likely because DLS measures hydrodynamic diameter, while AFM measures particle size (55).

**Figure 6:**
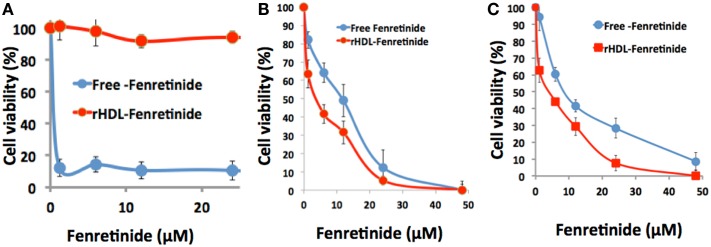
**Differential cytotoxicity of free fenretinide vs. the rHDL encapsulated drug against non-malignant, ARPE-19 cells (A) and two neuroblastoma cell lines SMS-KCNR (B) and SK-N-SH (C)**.

The impact of free FR vs. rHDL/FR on survival of the NB cell lines SK-N-SH and SMS-KCNR and the non-malignant ARPE-19 cell line are shown in Figure 6.

The cytotoxicity assessments of free FR vs. rHDL/FR toward the NB cell lines SK-N-SH and SMS-KCNR, calculated form the data shown in Figure 6, yielded respective half maximum inhibitory concentration (IC_50_) that were 2.8 and 2 times higher for free FR vs. the values for the rHDL encapsulated FR formulations (Table 1). On the other hand the IC_50_ value of Free FR for the retinal pigment epithelial cells (ARPE-19) was less than 40 times that of rHDL/FR. These observations suggest that when FR is delivered via rHDL vehicle, it’s toxicity to retinal pigment cells (and perhaps to other low SR-B1 expressor normal cells) is markedly reduced. These observations represent improvements of at least 80–112 times in *in vitro* therapeutic index, via encapsulation of FR into rHDL nanoparticles. These findings are also consistent with the selective impact of rHDL associated anti-cancer agents on malignant vs. non-malignant cells and tissues, reported earlier (34, 36).

**Table 1:** **Comparative IC_50_ doses of free and rHDL encapsulated FR on Neuroblastoma cells and retinal pigment epithelial cells**.

Cell type	IC_50_ free fenretinide (μM)	IC_50_ rHDL/fenretinide (μM)
ARPE-19	<1.2	>48
SMS-KCNR	14	5
SK-N-SH	7.2	3.5

**Figure 7:**
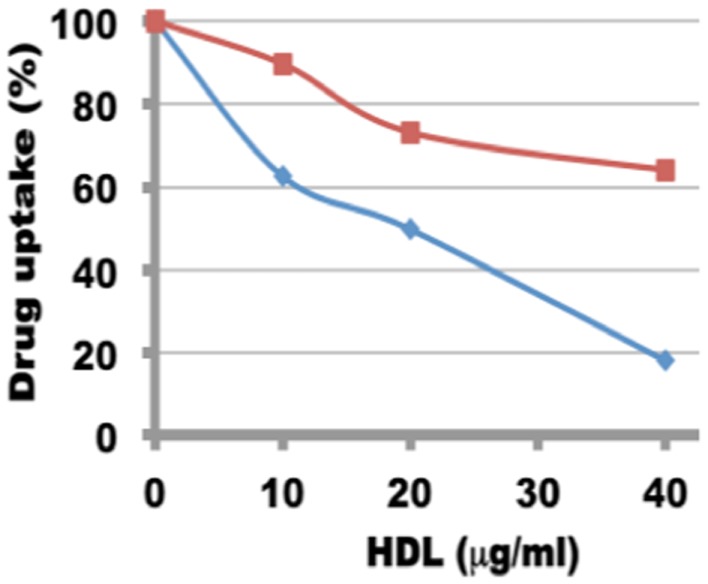
**Inhibition of uptake of ATRA and FR by in SMS-KCNR (neuroblastoma) cells from rHDL nanoparticles by human HDL**.

The uptake of both rHDL/ATRA and rHDL/FR nanoparticles by the NB cell line SMS-KCNR was investigated in the presence and absence of increasing amounts of human HDL (a competitor for the SR-B1 receptor) (36, 41). As shown in Figure 7, there was a gradual decrease in the uptake of ATRA from 89.7, 73, and 64.4% in response to the inclusion of 10, 20, and 40 μg/ml of human HDL in the incubation mix. The rHDL/FR particles showed an even sharper decrease in the uptake of FR from 62.65, 49.7, and 18.0% in response to the increasing amounts of human HDL. These studies indicate that the uptake of ATRA and FR from the rHDL particles is facilitated by the SR-B1 (HDL) receptor as shown for other anti-cancer agents by our earlier studies (36, 37, 41).

**Figure 8:**
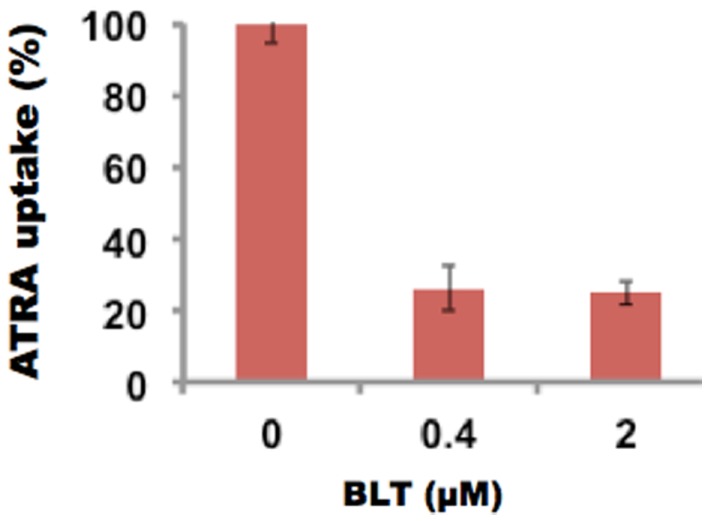
**Inhibition of ATRA uptake by SMS-KCNR (neuroblastoma) cells via the SR-B1 inhibitor BLT**.

The inhibition of uptake of ATRA was also studied in the presence of BLT-1, 2-Hexyl-1-cyclopentanone thiosemicarbazone, (BLT), a known inhibitor of SR-B1 receptor. At 0.4 μM BLT, the uptake of ATRA was reduced to 26%. These studies (Figure 8) further support the SR-B1 mediated mechanism of drug delivery via rHDL nanoparticles.

**Figure 9:**
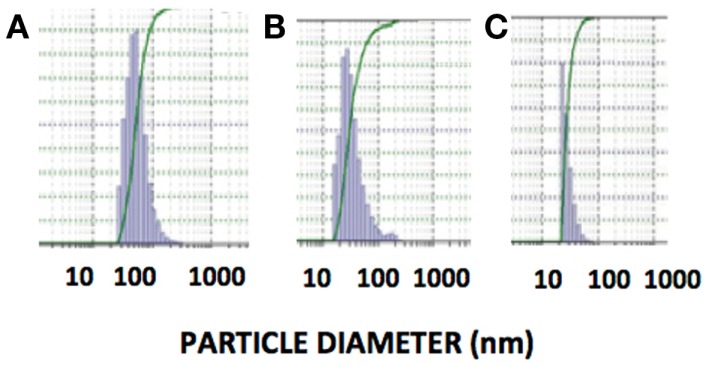
**Size distribution of rHDL/FR nanoparticles determined by dynamic light scattering after storage under different conditions**. **(A)** Storage at 4°C for 1 month, **(B)** Storage at −20°C for 1 month, **(C)** Post-Lyophilization.

Stability studies of rHDL-FR particles at 4°C, −20°C, and post-lyophilization resulted in retention of 89, 92, and 94.2% drug when stored for 1 month (Table 2). The particles appeared clear and uniform with minimal aggregation as indicated by the DLS patterns before (Figure 4B) and after storage (Figures 9A–C). These studies indicate that the preparations are considerably stable and non-leaky at the given conditions.

**Table 2:** **Percentage retention of FR in rHDL-FR nanoparticles when exposed to different storage conditions**.

Storage condition	Retention of FR (%)	Average diameter by DLS (nm)
4°C for 1 month	89	39.5
−20°C for 1 month	92	31.8
Lyophilization at −56°C	94.2	28.4

## Discussion

Systemic toxicity of drugs is a major limitation of the current chemotherapy approaches for treating cancer in general and HRNB tumors in particular (3–5, 56). This is an acute problem in pediatric oncology considering a limited tolerance for pain and discomfort among pediatric patients. Systemic toxicity is generally attributed to the “off target” impact of anti-cancer agents even when they are preferentially targeted to kill cancer cells and tumors (57–59). Evasion of systemic toxicity is thus paramount in the pediatric age group because their immature hepatic and renal systems may limit the dosages of anti-cancer drugs to be used during chemotherapy. At diagnosis, 50% of NB-bearing children exhibit metastatic disease with poor prognosis (7). There is thus an urgent need for novel therapeutic approaches with low off target toxicity for treating HRNB.

Since their discovery, both natural and synthetic retinoids have been known to play a pivotal role in manipulating cell and tissue differentiation. Although FR, a synthetic analog of ATRA, is the most studied anti-cancer retinoid its mechanism of action in controlling carcinogenesis and metastasis is considerably different from that of ATRA (60). The cytotoxic effect of FR is known to be due to the production of the sphingolipid, ceramide (61, 62) that acts as a signaling molecule regulating the differentiation, proliferation, and apoptosis. Ceramide build up in tumor cells is associated with the accumulation of reactive oxygen species (ROS), resulting in accelerated cell death *via* apoptosis and/or necrosis (63). Although ATRA is known to be a teratogen (64, 65), it has also been investigated as a differentiating agent to prevent the relapse of NB, subsequent to an intensive chemotherapy (66, 67). While FR and ATRA have both been shown to be effective as adjuvants in NB therapeutics, they both exhibit night blindness as a side effect (21, 66).

The aim of this project was to characterize the ATRA and FR containing rHDL nanoparticles and to evaluate the feasibility for enhancing the cytotoxic efficacy of FR against NB cells. In addition, the potential for eventual effective systemic administration of FR may be explored due to the improvement insolubility and bioavailability via inclusion of the drug in rHDL nanoparticles. In the present study, ATRA was used because of its physicochemical similarities with FR. Also availability of a tritium labeled isotope of ATRA enabled us to perform cellular uptake studies in the presence of BLT that was not possible with FR. Considering the teratogenic risks involved with the handling of ATRA, the specific manufacturer’s safety instructions were strictly followed throughout the studies. Two of the desirable features of an effective systemic formulation are to have both small and uniformly distributed particles. The PDI is a measure of the uniformity of size of nanoparticles and is based on the concept that scattering of light from small particles in a fluid media is a function of their diameter. For drug delivery, the smaller the PDI value, it is considered to be a more desirable characteristic of the formulation (49). For most nanoparticles the PDI value < 0.2 is recommended for intravascular use as drug carriers. In our initial studies, the ATRA preparation at 0.75 mg/ml initial drug concentration exhibited the particle diameter of 89 nm and the lowest PDI (0.05). Hence for further studies, 0.75 mg/ml initial drug concentration was chosen throughout our studies for the preparation of rHDL/ATRA and rHDL/FR nanoparticles. The size distribution of the rHDL formulations have shown reasonable homogeneity as indicated by the low PDI (<0.1). A single peak on DLS analysis for both rHDL/ATRA and rHDL/FR nanoparticles represents a relatively uniform distribution of the nanoparticles in solution. These patterns are consistent with earlier studies reported for rHDL valrubicin and rHDL paclitaxel nanoparticles (36, 40).

We studied the growth inhibitory concentrations of FR as a free drug and as a component of rHDL nanoparticles against a “retinal pigment epithelial cell line” ARPE-19. A comparative IC_50_ data analysis of NB cell lines and the retinal pigment (non-malignant) epithelial cell line indicates that when encapsulated into rHDL the ARPE-19 cells were markedly better protected against FR cytotoxicity vs. the impact of the free drug. These observations suggest that the rHDL drug delivery system may be effective in reducing the side effects of chemotherapy during the treatment of NB patients with conventional anti-cancer agents. These observations are consistent with effect of rHDL associated drug particles on malignant and non-malignant cells reported earlier with other drugs (34, 36).

The selective tumor uptake of drugs from rHDL nanoparticles via the SR-B1 receptor has previously been demonstrated for malignant prostate and ovarian cell lines (36, 41, 68). During the present studies, human HDL was added as a competitor to suppress uptake of FR and ATRA from the rHDL nanoparticles by NB cells (SMS-KCNR). These data (Figure 6) show that the NB cells tested are likely to overexpress the SR-B1 receptors, as it has been shown for nearly all other malignant cells and tumors (36, 41, 68). This observation is further supported by inhibition of drug uptake from the rHDL-ATRA nanoparticles by the NB cells in the presence of BLT, a chemical inhibitor of SR-B1 function (Figure 7). As mentioned previously, the estimation of FR concentration by spectrophotometry (A_340_) in the presence of BLT was not possible due to interference in the FR absorbance by BLT. Thus these experiments were conducted only with rHDL-ATRA particles. These observations taken together indicate that FR and ATRA when encapsulated in rHDL could be used as an alternative or adjuvant therapy for the treatment of NB patients resulting in enhanced therapeutic efficacy, with anticipated reduction in off target toxicity. Clearly, more advanced studies, especially evaluation of *in vivo* therapeutic efficacy and pharmacokinetics will be required before the rHDL model can be fully evaluated as a useful systemic therapeutic approach for treating NB patients.

Previous research has indicated that cancer cells have an enhanced expression of the SR-B1 receptor compared to that of normal cells (36, 41, 68–70). The rHDL drug delivery has been shown to be capable of selective delivery of anti-cancer agents via SR-B1 receptors, leaving normal cells (with either absent or low in SR-B1 expression) unharmed (36, 41, 68). Selective, tumor specific delivery of drugs would thus greatly enhance their therapeutic efficacy, *especially due to the anticipated reduced off target toxicity, (a major concern in pediatric oncology) when encapsulated into rHDL nanoparticles*. In addition due to the biocompatibility of rHDL nanoparticles, their clearance via the reticulo-endothelial system is likely to be prevented thus enhancing the residence time of the drug in the circulation.

### Translational significance

Fenretinide therapy has been employed against refractory NB or HRNB; however, due to its poor bioavailability and side effects it has been unsuccessful in phase II clinical trials. In the present report, we provide preliminary evidence that strongly favors the rHDL drug delivery system as a unique and effective tool for treating HRNB, including potential for minimizing side effects. This report also provides a conceptual background for designing clinical studies for evaluating other anti-cancer agents that are also anticipated to benefit from the selective delivery features of the rHDL drug delivery system during chemotherapy. This new approach, perhaps combined with other treatment modalities may be successful in improving the survival rates for HRNB.

## Conflict of Interest Statement

The authors declare that the research was conducted in the absence of any commercial or financial relationships that could be construed as a potential conflict of interest.
